# Assessing Nutrient Removal Kinetics in Flushed Manure Using *Chlorella vulgaris* Biomass Production

**DOI:** 10.3389/fbioe.2017.00043

**Published:** 2017-07-27

**Authors:** Pramod Pandey, Jun Shi

**Affiliations:** ^1^Department of Population Health and Reproduction, School of Veterinary Medicine, University of California Davis, Davis, CA, United States; ^2^Division of Agriculture and Natural Resources, University of California Cooperative Extension, Davis, CA, United States; ^3^State Key Laboratory of Pollution Control and Resources Reuse, College of Environmental Science and Engineering, Tongji University, Shanghai, China

**Keywords:** algal biomass production, flushed dairy manure, nutrient kinetics, treatment, biofuels

## Abstract

The utilization of dairy wastewater for producing algal biomass is seen as a two-fold opportunity to treat wastewater and produce algae biomass, which can be potentially used for production of biofuels. In animal agriculture system, one of the major waste streams is dairy manure that contains high levels of nitrogen and phosphorus. Furthermore, it is produced abundantly in California’s dairy industry, as well as many other parts of the world. We hypothesized that flushed manure, wastewater from a dairy farm, can be used as a potential feedstock after pretreatment to grow *Chlorella vulgaris* biomass and to reduce nutrients of manure. In this study, we focused on investigating the use of flushed manure, produced in a dairy farm for growing *C. vulgaris* biomass. A series of batch-mode experiments, fed with manure feedstock and synthetic medium, were conducted and corresponding *C. vulgaris* production was analyzed. Impacts of varying levels of sterilized manure feedstock (SMF) and synthetic culture medium (SCM) (20–100%) on biomass production, and consequential changes in total nitrogen (TN) and total phosphorus (TP) were determined. *C. vulgaris* production data (Shi et al., 2016) were fitted into a model (Aslan and Kapdan, 2006) for calculating kinetics of TN and TP removal. Results showed that the highest *C. vulgaris* biomass production occurs, when SMF and SCM were mixed with ratio of 40%:60%. With this mixture, biomass on Day 9 was increased by 1,740% compared to initial biomass; and on Day 30, it was increased by 2,456.9%. The production was relatively low, when either only SCM or manure feedstock medium (without pretreatment, i.e., no sterilization) was used as a culture medium. On this ratio, TN and TP were reduced by 29.9 and 12.3% on Day 9, and these reductions on Day 30 were 76 and 26.9%, respectively.

## Introduction

While conversion of algal biomass into biofuel is well reported, additional efforts are needed for identifying the pragmatic and less expensive feedstock (i.e., culture medium), which can lower the cost of algal biomass production, and helps in reducing environmental contamination as well as protecting ambient water (Yang et al., 2011; Borowitzka, 2013; Chiu et al., 2015; Van Wagenen et al., 2015). Here, we hypothesized that flushed dairy manure, which is abundantly produced in a dairy farm and easily available, can be potentially used for growing algal biomass such as *Chlorella vulgaris* and the biomass production can help in lowering excessive nutrients [total nitrogen (TN) and total phosphorus (TP)] in manure, which poses environmental challenges by increasing nutrients in ambient water. In previous published study (Shi et al., 2016), we have focused on evaluating the growth of *C. vulgaris*, however, the impacts of *C. vulgaris* on manure nutrient kinetics was not well analyzed. The goal of this study was to understand the nutrients removal from dairy manure by *C. vulgaris*.

Currently, substantial techno-economic challenges exist for making algal biomass-based biofuel as a practical alternative to petroleum-based fuels (Hu et al., 2012; Song et al., 2014; Zhou et al., 2014). One option, which has a potential to lower the cost of medium used for algal biomass production, is to exploit the use of wastewater streams produced in various treatment processes (Singh et al., 2011; Hu et al., 2012; Åkerström et al., 2014; Yadavalli et al., 2014; Van Wagenen et al., 2015). In this study, we focused on understanding the potential application of manure-based growth media for algae culture for producing algal biomass and reducing nutrients in dairy manure wastewater.

A number of wastewater streams, including industrial wastewater streams and municipal wastewater streams, have been tested at experimental scale to evaluate the feasibility of using wastewater effluent for algal biomass production. As an example, the effluent of anaerobic digesters, internal circulation reactor treating the waste of biotechnology production facility, was utilized as a feedstock for *Chlorella sorokiniana* production. The results showed that it can remove 99% ammonia and phosphate with biomass production of 1.33 g/L day (Van Wagenen et al., 2015). Another study used the municipal wastewater for *C. vulgaris* and *Planktothrix isothrix* production under monoalgal and co-cultures system (Silva-Benavides and Torzillo, 2012). Approximately 80% of nitrogen removal was achieved by the co-culture of *C. vulgaris* and *P. isothrix*. Similarly, the production of *Scenedesmus obliquus* was tested in multiple waste discharges, including fish pond discharges and municipal secondary settling tank discharges (Mandal and Mallick, 2011). The results showed that biomass yield ranged from 1.1 to 2.1 g/L in municipal secondary settling tank discharges and 0.6–2.0 g/L in fish pond discharges.

In addition to municipal waste (or industrial organic waste streams), animal waste streams generated in livestock industry could be a potential source for algal biomass production (Mulbry and Wilkie, 2001; Mulbry et al., 2008; Abreu et al., 2012; Kothari et al., 2012; Shi et al., 2016). However, additional studies are needed in order to prove the uses of manure for algal biomass production as a feasible alternative. Mixotrophic cultivation of *C. vulgaris* using industrial dairy waste as organic source showed enhanced algal biomass growth (Abreu et al., 2012). Final biomass concentrations and lipid productivity indicated the suitability of dairy wastewater for growing *C. vulgaris* (Abreu et al., 2012). While comparing the characteristics of *C. sorokiniana* CS-01 and UTEX 1230 strains, grown in various media including anaerobic digested effluent from cattle manure and in Bold’s Basal Medium (BBM), results showed that medium with anaerobic digester effluent from cattle manure produced comparable biomass growth to that in BBM. Biomass productivity was ≈280 mg/L.

Piggery effluent was also tested for growing *C. vulgaris* (Kumar et al., 2010). The use of digested piggery effluent as nutrient source to substitute for mineral nutrients resulted in biomass-specific growth rate of 0.34 day^−1^. The study concluded that high production of *C. vulgaris* can be achieved in short time by feeding digested effluent of piggery effluent. The poultry litter waste was also found to be a suitable medium for microalgal growth. Algal strains of *Chlorella minutissima, Chlorella sorokiniana*, and *Scenedesmus bijuga* are reported to grow on anaerobically digested poultry litter. A biomass productivity of 76 mg/L was observed in a medium, which was a mixture of poultry litter anaerobic digester effluent and deionized water (Singh et al., 2011). The suitability of poultry litter for growing *S. obliquus* for lipid production has also been reported (Mandal and Mallick, 2011).

In animal agriculture system, one of the major waste streams in the USA is dairy manure. As an example, more than 20 million tons of dairy manure is produced annually in the USA and the potential of this manure is underutilized (Smith et al., 2016). The United State Department of Agriculture estimates that there are more than 5.2 million cattle and calves in California, which produces million tons of manure each year. Currently, majority of this manure is applied in the cropland as fertilizer (Pandey et al., 2015). Identifying the alternative uses of dairy manure that has a potential to increase the value of manure will likely to improve farm incomes as well as reduce the environmental problems associated with dairy manure such as excess influx of nutrients into ambient water.

The hypothesis of this study was that the pretreated flushed dairy manure could possibly be used as a suitable medium to grow algal biomass and reduce the nutrients of manure wastewater produced in a dairy farm. This study is built on a previous study (Shi et al., 2016), which explored the use of flushed manure to evaluate the growth of *C. vulgaris*. To extend the previous study, here we have evaluated the nutrient recovery kinetics as a result of biomass production. A model (Aslan and Kapdan, 2006) was applied to estimate the TN and TP kinetics under *C. vulgaris* production in dairy manure. The primary objective of the study are as follows: (1) understand the change in TN and TP corresponding to biomass production in a series of experiments using pretreated and untreated flushed manure and (2) calculate TN and TP removal rate, and kinetics of nutrient recovery under *C. vulgaris* growth in feedstock, where flushed manure was supplemented with various levels of synthetic media.

## Materials and Methods

### Dairy Wastewater Sample

Lagoon manure water samples were collected from lagoons located in dairy farms in Merced, Glenn, and Tulare Counties of California Central Valley, USA. These dairy facilities houses ≈3,000–5,000 milking and non-milking dairy cows. In general, dairy farms in California Central Valley use flushed system that produces flushed liquid manure (FLM), which is a mixture of feces and urine, parlor wash-off, and pre-milking wash pen drainage. Subsequently, the FLM passes through a separator to fractionate liquid and solids. Liquid portions go into lagoons and solid fractions are piled, which is eventually used either as fertilizers or bedding materials. After collections, samples were transported using coolers and subsequently samples stored at 4°C prior to starting the experiment. The FLM samples were centrifuged (ThermoFisher Sci.: Sorvall Legend X1R) at 10,000 rpm for 15 min to remove unwanted solids and fibers. The supernatant was used as manure feedstock medium (MFM). Furthermore, a subset of the MFM was sterilized at 121°C for 15 min to inactivate manure-borne microbial population and this medium was termed as sterilized manure feedstock (SMF).

### Synthetic Medium

In addition to the manure-based medium (MFM and SMF), synthetic culture medium (SCM) was prepared. The SCM includes 958 mL of distilled water, 0.25 g of NaNO_3_, 0.075 g of K_2_HPO_4_⋅3H_2_O, 0.075 g of MgSO_4_⋅7H_2_O, 0.025 g of CaCl_2_⋅2H_2_O, 0.175 g of KH_2_PO4, 0.025 g of NaCl, 40 mL of soil extract solution, 0.005 g of FeCl_3_⋅6H_2_O, 1 mL of Fe-EDTA solution, and 1 mL of A5 solution. The soil extract solution was prepared with 200 g of unfertilized garden soil and 1,000 mL of distilled water. After mixing garden soil with distilled water, the mixture was heated in water bath for 3 h and cooled for 24 h. As a filtering step, the solution was filtered using 0.45 µm filter and supernatant was used as a soil extract solution. The Fe–EDTA solution was prepared by mixing 50 mL distilled water, 1 g of Na_2_EDTA, 81 mg of FeCl_3_⋅6H_2_O, and 50 mL of 0.1 N HCl. The A5 solution includes 2.86 g/L of H_3_BO_3_, 1.86 g/L of MnCl_2_⋅4H_2_O, 0.22 g/L of ZnSO_4_⋅7H_2_O, 0.39 g/L Na_2_MoO_4_⋅2H_2_O, 0.08 g/L of CuSO_4_⋅5H_2_O, and 0.05 g/L of Co(NO_3_)_2_⋅6H_2_O. The SCM was autoclaved and stored at 4°C prior to use it.

### Experiment Design

The growth of *C. vulgaris* (UTEX-2714) was assessed in MFM, SMF, and SCM media. Furthermore, the growth was also investigated in mixed environments MFM:SCM and SMF:SCM ratios of 20:80, 40:60, and 70:30. The growth experiment was conducted in 500 mL conical flasks at a constant temperature (25 ± 1°C). The strain of *C. vulgaris* was obtained from the Culture Collection of Algae, University of Texas, Austin, TX, USA. The pre-cultured *C. vulgaris* [optical density (OD) 680 ≈ 0.355] was inoculated into 500 mL conical flasks with a proportion of 20% (v/v) under sterile conditions. To avoid the potential ambient contamination, the experiments were conducted in biological controlled environment [i.e., inside a bio-safety cabinet level II (SterilGARD Hood, the Baker Company Inc.)]. Additional details of experiments are described elsewhere (Shi et al., 2016). In brief, to design the experiment environment, a bio-safety cabinet was converted into a photo-bioreactor. The light was controlled using two 4 ft. T12 40-W Cool White Supreme (4,100 K) Alto Linear Fluorescent Light Bulb with brightness of 2,600 lm. The temperature was controlled using a heating/cooling tower (Dyson-AM09 Fan, Model: 302198-01). The cultivation was carried out for 9 and 30 days in 12 h light and 12 h dark conditions. The first 9 days, samples were collected regularly to monitor biomass growth and nutrient recovery. The light and dark conditions were controlled by using an electric timer (CUTNSTK624, Prime). Intermittent shaking by hand of the culture flasks (twice a day) was provided for the first week of cultivation only and, subsequently, the flasks were left undisturbed (i.e., no shaking was provided).

### Analysis

#### Biomass Analysis

The growth of algae was estimated by measuring the OD of the samples containing *C. vulgaris* at 680 nm wavelength. The instruments and method used for OD measurement is presented previously (Shi et al., 2016). For algal biomass calculation, approximately a 10 mL of growth medium was centrifuged at 8,000 rpm for 10 min and the pellets were washed twice with distilled water for removing the excess salts and solids. Subsequently, the pellets were resuspended in distilled water and filtered through a pre-weighted 47 mm membrane filter (HAWG047S6, Millipore). The filters with *C. vulgaris* biomass were dried overnight at 60°C and weighted. An empirical equation (Eq. 1) was developed to relate *C. vulgaris* OD 680 values and dry biomass, which resulted in a *R*^2^ of 0.98 and was used further for calculating the biomass from OD 680 readings

(1) $$B_{m}=0.3386\cdot \text{OD}680,$$

where *B_m_* is biomass dry weight (g/L).

#### Chemical Analysis

Total nitrogen and TP concentrations were analyzed for estimating the TN and TP losses in manure due to biomass growth. Samples withdrawn from flasks every 2 days were centrifuged at 10,000 *g* for 15 min to separate algae. Standard method 10071 and 8190 was applied for the TN and TP analysis. The HACH reagents and a spectrophotometer (UV/VIS Ultraviolet Photometer, 350–1,020 nm) were used for the analysis.

#### Model Development

To estimate the TN and TP removal rates, we used the removal rate equations (Aslan and Kapdan, 2006)

(2) $$R_{r}=-\frac{S_{i}-S_{t}}{T_{i}-T_{t}},$$

where *R_r_* represents the substrate removal rate, *S_i_* is the initial substrate concentrations as TN or TP, *S_t_* is the corresponding substrate concentration at *T_t_*. The *T_t_* is the time when concentration of the substance did not change. The initial substrate removal rate is the slope of time versus effluent concentration at time *T_t_*. The specific rate of substrate removal (*R*_sr_) was estimated as *R*_sr_ = *R_r_*/(*B_m_*)*_i_*. The Michaelis–Menten kinetics was used to calculate kinetic coefficients (*K_m_*), saturation constant (*k*), and reaction rate constant (*R*) (Eq. 3) (Aslan and Kapdan, 2006)

(3) $$R=\frac{R_{\text{mr}}S_{e}}{K_{m}+S_{e}},$$

where *R*_mr_ is the maximum substrate removal rate and *S_e_* is the effluent substrate concentration. The substrate concentration corresponds to half reaction rate gives the saturation constant. The author (Aslan and Kapdan, 2006) proposed equations for calculating the initial substrate concentrations and the initial substrate removal rates, which were used for estimating *R*_si_

(4) $$R_{\text{si}}=\frac{R_{\text{mi}}S_{i}}{K_{m}+S_{i}}.$$

Considering *R*_mi_ = *k*·*X_i_*, which is the maximum initial substrate removal rate. The modified version of Eq. 4 was proposed for *R*_si_ estimation (Shi et al., 2016)

(5) $$R_{\text{si}}=\frac{kX_{i}S_{i}}{K_{m}+S_{i}}$$

where *k* is the reaction rate constant (day^−1^), *X_i_* is the initial biomass concentration for algae. The specific substrate removal (*R*_sr_) rate was calculated as:

(6) $$R_{\text{sr}}=\frac{R_{\text{si}}}{X_{i}}=\frac{kS_{i}}{K_{m}+S_{i}}.$$

The slope (*K_m_*/*k*) and intercept (1/*k*) was estimated (Aslan and Kapdan, 2006) by plotting a linear line between 1/*R*_sr_ versus 1/*S_i_*

(7) $$\frac{1}{R_{\text{sr}}}=\frac{1}{k}+\frac{K_{m}}{k}\frac{1}{S_{i}}.$$

Yield coefficient for TN and TP removal was calculated as:

(8) $${\left(B_{m}\right)}_{f}-{\left(B_{m}\right)}_{i}=Y_{N}\left({\text{TN}}_{i}-{\text{TN}}_{f}\right),$$

(9) $${\left(B_{m}\right)}_{f}-{\left(B_{m}\right)}_{i}=Y_{P}\left({\text{TN}}_{i}-{\text{TN}}_{f}\right),$$

where (*B_m_*)*_f_* is the final biomass concentration (mg/L), (*B_m_*)*_i_* is the initial biomass concentration (mg/L) (Aslan and Kapdan, 2006). The TN*_i_* and TN*_f_* are the initial and the final TN concentrations (mg/L), respectively. Slope of plot between [(*B_m_*)*_f_* − (*B_m_*)*_i_*] and [TN*_i_* − TN*_f_*] resulted yield coefficient for TN, i.e., *Y_N_* (mg *B_m_*/mg TN). Similar approach can be implemented for the TP.

## Results

### Change in TN and TP Concentrations

**Figure 1:**
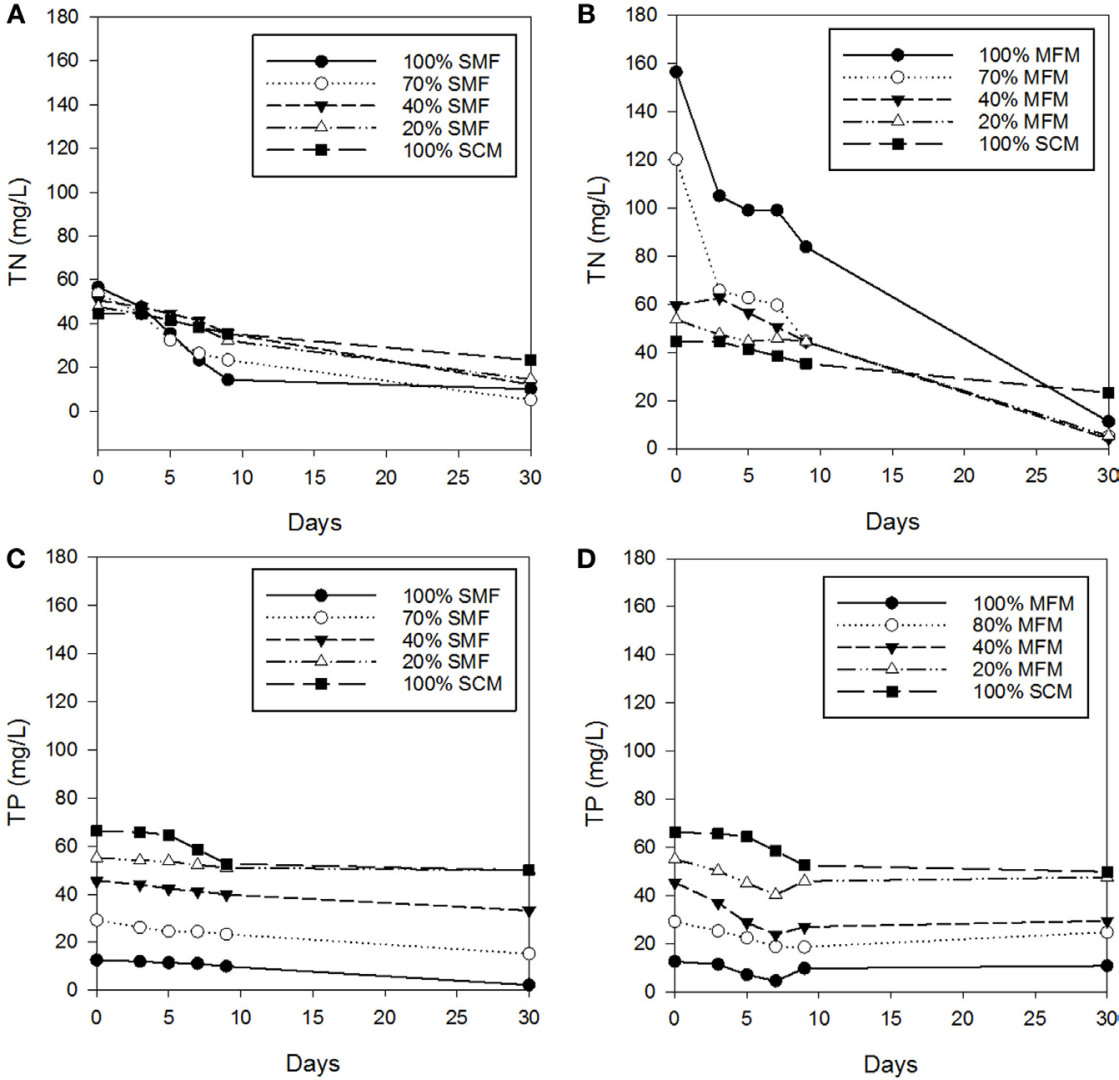
Changes in total nitrogen (TN) and total phosphorus (TP) concentrations: **(A)** TN concentrations under sterilized manure feedstock (SMF) and synthetic culture medium (SCM) ratio; **(B)** TN concentrations under manure feedstock medium (MFM) and SCM ratios; **(C)** TP concentrations under SMF and SCM ratios; and **(D)** TP concentrations under MFM and SCM ratios.

The change in TN and TP was evaluated for a various levels of sterilized (pretreated) manure feedstock (i.e., SMF), untreated MFM (i.e., MFM), and SCM. The experiment lasted for total of 30 days, however, during the first 9 days, the observations were made daily and, subsequently, the measurements were made on Day 30. With regard to algal biomass harvesting, a growth cycle of 10 days is often used for biomass productivity estimation (Shi et al., 2016). Results of TN and TP changes (Figure 1) under various growth conditions showed that nutrient recovery under untreated manure condition was different than that of pretreated manure condition.

The dynamics of TN and TP change in SMF and MFM with comparison to SCM is shown in Figure 1. The TN removals (in sterilized manure) at ratio of 100% SMF, 70% SMF, 40% SMF, 20% SMF, and 100% SCM were 74.9, 56.5, 29.9, 32.2, and 20.4% for TN*_i_* = 44.5–56.6 mg/L under 9 days cultivation. Under 30 days cultivation period, the TN removals were 82.4, 90.4, 76.0, 69.4, and 47.6%, respectively. When pretreated (i.e., sterilized) manure was used as medium, the TN removal for 30 days cultivation period was higher than that for 9 days cultivation period.

Under non-sterilized (untreated) manure feedstock condition, the TN removals at ratio of 100% MFM, 70% MFM, 40% MFM, 20% MFM, and 100% SCM were 46.4, 63.0, 25.4, 17.0, and 20.4%, respectively, for TN*_i_* = 53.6–156.4 mg/L under 9 days cultivation period. For the similar feedstock condition, on 30 days of culture, the removal rates were 92.9, 95.7, 93.1, 90.1, and 47.6%, respectively, for corresponding ratios. Similar to sterilized condition, the TN removal under untreated manure feedstock (i.e., no pretreatment) condition was higher for 30 days culture period than that for 9 days culture period. However, the TP concentrations remain consistent, i.e., the concentration of TP on Days 9 and 30 was comparable under non-pretreatment condition (Figure 1).

During the first 9 days culture period under SMF condition, the removals of TP for 100% SMF, 70% SMF, 40% SMF, 20% SMF, and 100% SCM were 20.9, 20.0, 12.3, 7.4, and 20.8%, respectively. The removals of TP in 30 days culture for these ratios were 82.4, 47.8, 26.9, 9.6, and 24.7%, respectively. For non-sterilized manure under 9 days culture period, the TP was removed by 22.8, 36.3, 40.6, 16.6, and 20.8% for 100% MFM, 70% MFM, 40% MFM, 20% MFM, and 100% MFM, respectively. Compared to non-sterilized manure condition, the TP removal in sterilized manure condition was slightly more consistent (Figures 1C,D) and linear.

### Biomass Production and Nutrient Removal Rates

**Figure 2:**
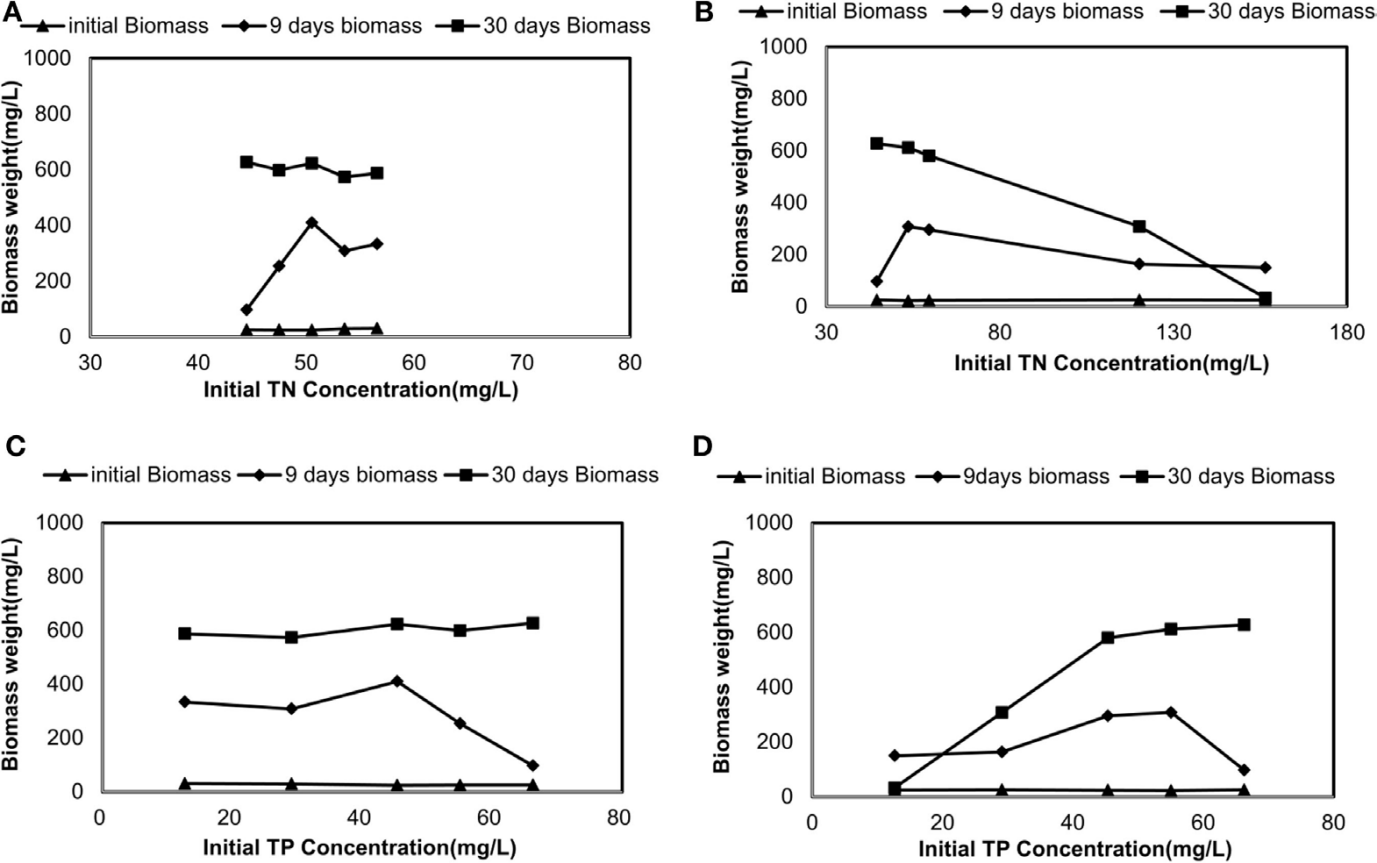
Changes in dry biomass weight corresponding to initial total nitrogen (TN) and total phosphorus (TP) concentrations: **(A)** TN-driven dry biomass weight under sterilized manure feedstock (SMF) and synthetic culture medium (SCM) ratios; **(B)** TN-driven dry biomass weight under manure feedstock medium (MFM) and SCM ratios; **(C)** TP-driven dry biomass weight under SMF and SCM ratios; and **(D)** TP-driven dry biomass weight under MFM and SCM ratio.

Biomass production corresponding to initial TN concentration is shown in Figures 2A,B. The change in biomass production corresponding to the TP is shown in Figures 2C,D. After seeding culture medium with *C. vulgaris*, the initial biomass concentrations did vary from 24 to 30 mg/L. In sterilized manure, the biomass production at the end of 9 days incubation period was 334, 308.6, 410.5, 253.7, and 96.9 mg/L for 100% SMF, 70% SMF, 40% SMF, 20% SMF, and 100% SCM, respectively. Under 30 days culture period for these conditions, the biomass production was 587.6, 573.4, 623.5, 598.8, and 627.3 mg/L, respectively, on Day 30.

The biomass production under non-sterilized medium was slightly lower than that under sterilized medium. During 9 days culture period, biomass reached to 150.0, 163.6, 295.7, 308.2, and 96.9 mg/L for 100% MFM, 70% MFM, 40% MFM, 20% MFM, and 100% SCM, respectively. The biomass production for the similar condition was increased on Day 30 (except for 100% MFM). The reduced biomass on Day 30 for 100% MFM was attributed to the decay of biomass growth. The biomass production for 30 days (under untreated manure) at 70% MFM, 40% MFM, 20% MFM, and 100% SCM were 308.2, 580.2, 612.0, and 627.3 mg/L, respectively. In general, the final biomass content in SMF medium increased with the increase in TN*_i_* = 44.5 mg/L to TN*_i_* = 56.6 mg/L and TP*_i_* = 12.7 mg/L to TP*_i_* = 66.3 mg/L. Similarly, the final biomass content in MFM medium was also increased with the increase in TN*_i_* = 44.5 mg/L to TN*_i_* = 156.4 mg/L and TP*_i_* = 12.7 mg/L to TP*_i_* = 66.3 mg/L (Figure 2).

### Batch Kinetic Coefficients

**Figure 3:**
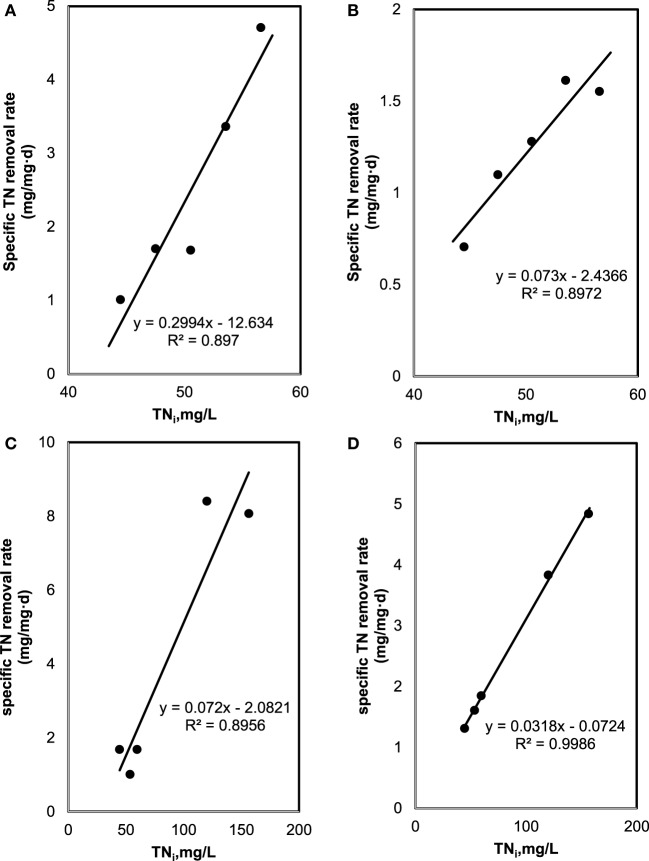
Total nitrogen (TN) removal: **(A)** effect of initial TN on specific TN removal rate under sterilized manure feedstock (SMF) and synthetic culture medium (SCM) ratios in 9 days cultivation; **(B)** effect of initial TN on specific TN removal rate under SMF and SCM ratios in 30 days cultivation; **(C)** effect of initial TN on specific TN removal rate under manure feedstock medium (MFM) and SCM ratios in 9 days cultivation; and **(D)** effect of initial TN on specific TN removal rate under MFM and SCM ratios in 30 days cultivation.

**Figure 4:**
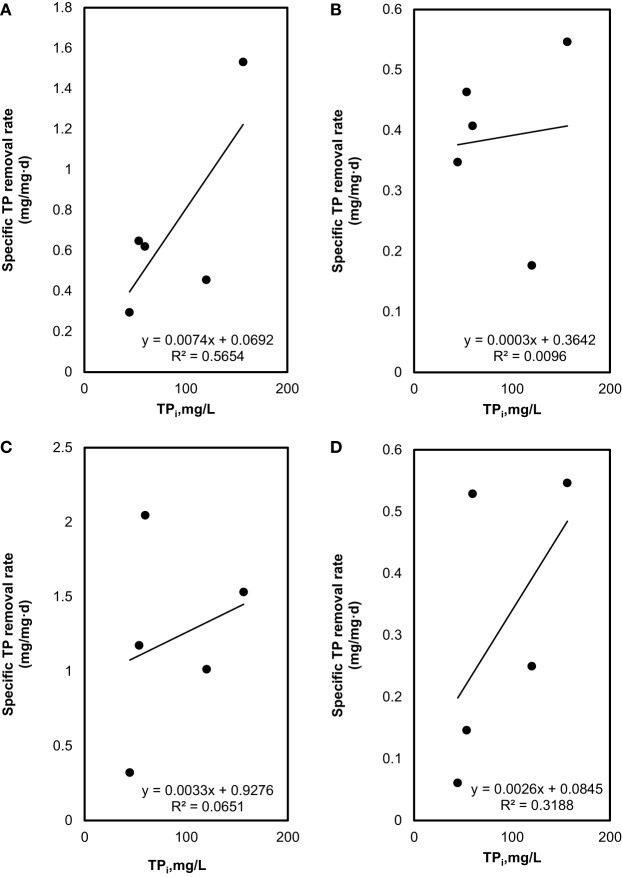
Total phosphorous (TP) removal: **(A)** effect of initial TP on specific TP removal rate under sterilized manure feedstock (SMF) and synthetic culture medium (SCM) ratios in 9 days cultivation; **(B)** effect of initial TP on specific TP removal rate under SMF and SCM ratios in 30 days cultivation; **(C)** effect of initial TP on specific TP removal rate under manure feedstock medium (MFM) and SCM ratios in 9 days cultivation; and **(D)** effect of initial TP on specific TP removal rate under MFM and SCM ratios in 30 days cultivation.

The specific TN and TP removal rate for varying TN and TP concentration are shown in Figures 3 and 4. The removal rate was increased with an increase in the initial TN concentration. The maximum removal rate was 4.7 mg/mg day in the SMF media and 8.1 mg/mg day in the MFM media. The maximum specific TP removal rates (Figure 4) were 0.65 mg/mg day and 2.05 mg/mg day in SMF and MFM medium, respectively. As shown in Figure 3, the relationship between specific TN removal rate and initial TN concentration was linear (*R*^2^ value varied from 0.89 to 0.99). However, the relationships between specific TP removal rate and initial TP concentrations were unpredictable and the linear fit resulted in relatively poor *R*^2^ values, which varied from 0.009 to 0.56.

**Figure 5:**
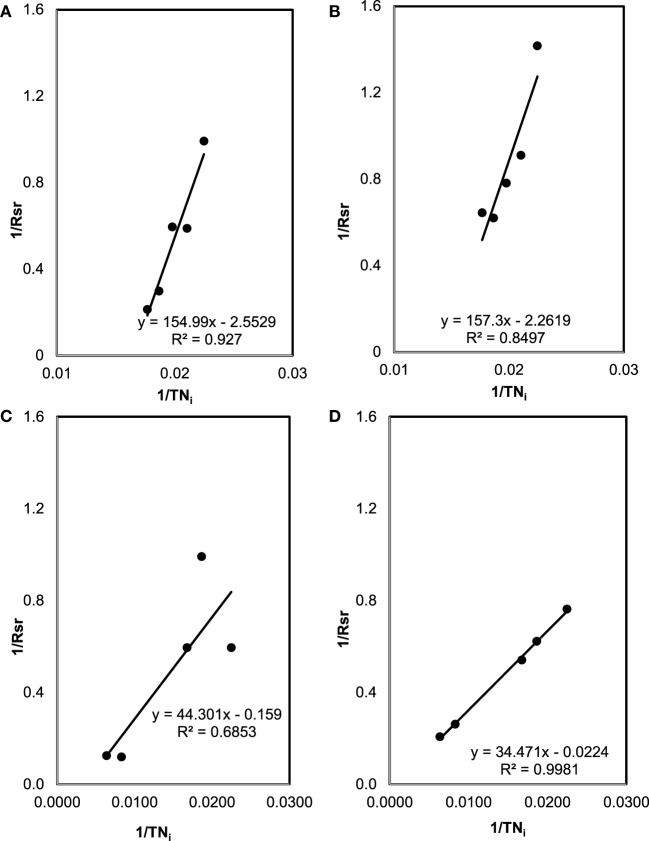
The plots between 1/*R*_sr_ versus 1/TN*_i_* used for the estimation of kinetic coefficients (*K_m_* and *k*) for total nitrogen (TN) removal: **(A)** sterilized manure feedstock (SMF) and synthetic culture medium (SCM) for 9 days cultivation; **(B)** SMF and SCM for 30 days; **(C)** manure feedstock medium (MFM) and SCM for 9 days; and **(D)** MFM and SCM for 30 days.

The plots between 1/*R*_sr_ versus 1/TN*_i_* are shown in Figure 5. From the slope and intercept of best fit line of these plots, kinetic coefficients of TN removal were determined. While *k* of 0.39 (mg TN/mg Biomass day) and *K_m_* of 60.7 mg/L (*R*^2^ = 0.93) were for 9 days cultivation period, *k* = 0.44 (mg TN/mg Biomass day) and *K_m_* = 69.5 mg/L (*R*^2^ = 0.85) were for 30 days for SMF experiment (i.e., pretreated condition). Under untreated manure feedstock condition (for MFM media), kinetic coefficients of TN removal were determined as *k* = 6.29 (mg TN/mg Biomass day) and *K_m_* = 278.6 mg/L (*R*^2^ = 0.88) for 9 days cultivation and *k* = 44.6 (mg TN/mg Biomass day) and *K_m_* = 1,538.9 mg/L (*R*^2^ = 0.88) for 30 days culture period.

Similarly, the coefficients for TP removal in SMF media were found as *k* = 2.0 (mg TP/mg Biomass day) and *K_m_* = 190.8 mg/L (*R*^2^ = 0.42) for 9 days; and *k* = 0.92 (mg TP/mg Biomass day) and *K_m_* = 0.25 mg/L (*R*^2^ = 0.16) for 30 days culture period. For untreated manure feedstock (i.e., MFM media), kinetic coefficients of TP removal were determined as *k* = 102.0 (mg TP/mg Biomass day) and *K_m_* = 0.40 mg/L (*R*^2^ = 0.37) for 9 days; and *k* = 106.4 (mg TP/mg Biomass day) and *K_m_* = 0.09 mg/L (*R*^2^ = 0.54) for 30 days.

## Discussion

Table 1 shows the descriptive statistics of biomass growth and nutrient removal rates under various treatment environments. Removal percentages of TN and TP, and percentages increase in biomass for SMF and FMF conditions are shown in Table 1. The removal rate and increase in biomass percentages were estimated for Day 9 and Day 30 and the calculations are based on initial and final concentrations. The TN removal for Day 9 cultivation varied from 29.9 to 74.9% under pretreated manure feedstock conditions, depending on the ratio between SMF and SCM. Under untreated condition, TN removal varied from 17 to 63%. The higher TN removal under pretreatment condition can be attributed to sterilization process, which may help the *C. vulgaris* use manure nitrogen efficiently. However, over the time (on Day 30), the nitrogen removal was higher for non-sterilized condition (i.e., untreated manure feedstock, MFM), which indicates that the consumption of untreated manure TN by algae was increased over the time.

**Table 1:** Descriptive statistics of nutrients and *Chlorella vulgaris* biomass growth in manure-based medium.

Feedstock descriptions/parameters		Minimum	Maximum	Average	SD	% Change (+/−)	
						Day 9	Day 30
**100% Sterilized manure feedstock (SMF)**							
SMF + synthetic culture medium (SCM)	Total nitrogen (TN) (mg/L)	10	56.6	31.2	18.6	−74.9	−82.4
	Total phosphorous (TP) (mg/L)	2.2	12.7	9.9	3.9	−20.9	−82.4
	Biomass weight (mg/L)	30.5	587.6	232.7	166.3	+1,215.6	+1,827.8
Manure feedstock medium (MFM) + SCM	TN (mg/L)	11.2	156.4	92.4	46.9	−46.4	−92.9
	TP (mg/L)	4.6	12.7	9.4	3	−22.8	−14.4
	Biomass weight (mg/L)	24.7	157.5	115.6	50.8	+527.4	+90.4

**70% SMF**							
SMF + SCM	TN (mg/L)	5.1	53.6	30.9	17.0	−56.5	−90.4
	TP (mg/L)	15.2	29.1	23.8	4.7	−20	−47.8
	Biomass weight (mg/L)	29.7	573.4	220.1	159.8	+1,150	+1,868.6
MFM + SCM	TN (mg/L)	5.1	120.1	59.6	37.2	−63.0	−95.7
	TP (mg/L)	18.6	29.1	23.2	4.1	−36.3	−15
	Biomass weight (mg/L)	25.4	382.7	144.8	91.9	+556	+1,406.7

**40% SMF**							
SMF + SCM	TN (mg/L)	12.1	50.5	38.6	14.0	−29.9	−76.0
	TP (mg/L)	33.1	45.4	40.9	4.3	−12.3	−26.9
	Biomass weight (mg/L)	24.4	623.5	284.3	180.4	+1,740.3	+2,456.9
MFM + SCM	TN (mg/L)	4.1	62.6	46.3	21.7	−25.4	−93.1
	TP (mg/L)	23.8	45.4	31.9	7.9	−40.6	−35
	Biomass weight (mg/L)	23.7	576.1	230.1	148.2	+1,177.1	+2,330

**20% SMF**							
SMF + SCM	TN (mg/L)	14.5	47.5	36.4	12.0	−32.2	−69.4
	TP (mg/L)	49.8	55.1	52.6	2	−7.4	−9.6
	Biomass weight (mg/L)	24.7	598.8	204.4	156.3	+1,084.9	+2,321.9
MFM + SCM	TN (mg/L)	5.3	53.6	40.2	17.4	−17.0	−90.1
	TP (mg/L)	40.3	55.1	47.4	5	−16.6	−13.6
	Biomass weight (mg/L)	22.4	566.3	227.3	150.7	+1,387.9	+2,433.3

**100% SCM**							
SCM	TN (mg/L)	23.3	44.5	37.9	8.0	−20.4	−47.6
	TP (mg/L)	49.9	66.3	59.6	7.1	−20.8	−24.7
	Biomass weight (mg/L)	26.4	309.9	88.5	82.1	+301.3	+1,120

The TN removal for Day 30 under SMF conditions varied from 69.4 to 90.4% and for MFM condition, it varied from 90.1 to 95.7% (Table 1). This indicates that for shorter growth period (≈10 days), sterilization of manure (pretreatment) could be a better approach for TN removal as well as biomass production. As an example, under sterilized condition (pretreatment) on Day 9, biomass weight was increased from 1,084.9 to 1,740.3% for various SMF and SCM ratios. Under untreated condition, biomass weight on Day 9 was increased only from 527.4 to 1,387.9% for various MFM and SCM ratios (Table 1). Under prolonged growth period (30 days), the biomass weight was increased by 1,827.8–2,456.9% under pretreatment condition and 90.4–2,433.3% under untreated conditions. When only manure feedstock (pretreated and untreated) was used for algal biomass production (no mixing with SCM), the biomass weight was considerably higher for pretreated manure condition than that for untreated condition under both growth periods (Day 9 and Day 30) (Table 1). While this preliminary provides important information in terms of nutrient removal from dairy manure by *C. vulgaris*, additional studies are needed to strengthen the results of this study and also to test the feasibility of using manure for producing *C. vulgaris* at large scale.

**Figure 6:**
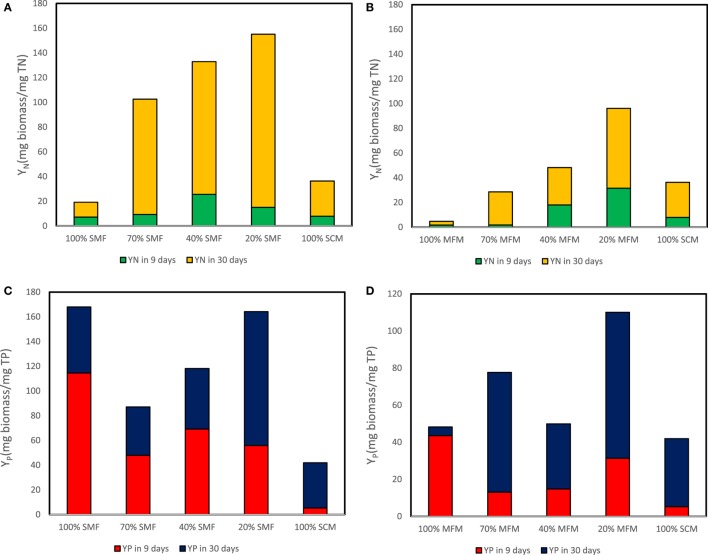
Yield coefficient for total nitrogen (TN) and total phosphorous (TP) removal: **(A)** yield coefficient for TN removal sterilized manure feedstock (SMF) and synthetic culture medium (SCM) ratios; **(B)** yield coefficient for TN removal under manure feedstock medium (MFM) and SCM ratios; **(C)** yield coefficient for TP removal under SMF and SCM ratios; and **(D)** yield coefficient for TP removal under MFM and SCM ratios.

The highest *Y_N_* in 9 days was determined as 25.5 (mg biomass/mg TN) at ratio of 40% SMF and 31.5 (mg biomass/mg TN) at ratio of 20% MFM media. For 30 days cultivation, the maximum yield coefficient for TN (i.e., *Y_N_*) reached 140.1 (mg biomass/mg TN) at 20% SMF and 64.7 (mg biomass/mg TN) at 20% MFM (Figures 6A,B). The highest *Y_P_* in 9 days were 31.5 (mg biomass/mg TP) at 20% SMF and 114.5 (mg biomass/mg TP) at ratio of 100% MFM. For 30 days cultivation, the maximum yield coefficient for TP reached 108.3 (mg biomass/mg TP) at 20% SMF and 78.7 (mg biomass/mg TP) at 20% MFM (Figures 6C,D).

In summary, the ranking of various media composition for TN removal (higher to lower) for SMF media was 40% SMF > 20% SMF > 70% SCM > 100% SMF > 100% SCM. For MFM media, the TN removal ranking was 20% SMF > 40% SMF > 100% SCM > 70% MFM > 100% MFM. The ranking (higher to lower) for removal of TP in SMF media was found as 100% SMF > 40% SMF > 20% SMF > 70% SMF > 100% SCM. This TP removal ranking for MFM media was found to be 100% MFM > 20% MFM > 40% MFM > 70% MFM > 100% SCM.

Similar to this finding, previous studies have shown that nutrient recovery changes considerable with feedstock (or growth medium), treatment conditions, and algae species. As an example, nitrogen and phosphorus recovery in piggery effluent by *C. vulgaris* is reported 100 and 78%, respectively (Kumar et al., 2010). Previous study (Zhu et al., 2013) reported 81 and 100% of nitrogen and phosphorus recovery by *Chlorella zofingiensis* in piggery wastewater. The growth of *S. obliquus* in urban wastewater resulted in nitrogen and phosphorus recovery by 100 and 98% (Martinez et al., 2000). The recovery of nitrogen and phosphorus in urine sample by the growth of *Spirulina platensis* is reported by 99% (each) (Yang et al., 2008). The recovery of nitrogen and phosphors in municipal wastewater by *C. minutissima* were found to be 30 and 41%, respectively (Bhatnagar et al., 2010). Another study also reported that the growth of *Chlorella* sp. in dairy waste water can recover nitrogen and phosphorus by 85 and 100% (Lu et al., 2015). This study suggests that TN and TP of flushed dairy manure can be reduced by algal biomass considerably depending on the treatment conditions. Furthermore, flushed manure can be applied as a growth (culture) medium for producing the algal biomass. For the shorter growth cycle (≈10 days growth period), pretreatment of manure (i.e., sterilization of manure) can be a preferred method for producing higher biomass in limited time and sterilization process can also help on eliminating unwanted microbial population/pathogen in manure.

## Conclusion

To improve the understanding of nutrient recovery using *C. vulgaris* from flushed dairy manure, here we performed a series of preliminary batch experiments for algal biomass production. The biomass production of *C. vulgaris* in a series of media, which includes synthetic media and manure mix, was used to evaluate nutrient kinetics. Results showed that nitrogen recovery was more consistent compared to phosphorus. The recovery of nitrogen and phosphorus changed with feedstock characteristics. In general, the growth of *C. vulgaris* in manure-based media resulted in reductions of nitrogen and phosphorus. The removal of TN was more consistent than the removal of TP. For Day 9 cultivation period, the TN removal varied from 29.9 to 74.9% under sterilized (pretreated manure) feedstock conditions. Under untreated manure condition, TN removal varied from 17 to 63%. This indicates that for shorter growth period (≈10 days), sterilization of manure (pretreatment) could be a better approach for TN removal as well as biomass production. These preliminary outcomes indicate that dairy manure can be utilized with certain pretreatment such as sterilization as a potential media for growing algal biomass. Additional studies are needed further to improve the findings of this study and understand the applicability of manure-based feedstock for algal biomass production at large scale, and its effects on dairy manure nutrient removal. The proposed method can reduce the cost of algal biomass production and lower the demand of chemical-based growth media. As manure is abundantly available, the process can result in a higher amount algal biomass production at low cost. This method can further assist in reducing the nutrients of manure, which otherwise would cause environmental pollution such as excessive nutrient concentrations in ambient water.

## Author Contributions

Experiment for use of animal waste for algal biomass production was conceptualize by PP and experiment setup was designed by PP with the help of JS. Manure feedstock from dairy farm was collected by PP and SCM media was prepared by JS. Experiment was conducted by JS and data were generated by JS. The observation and modeling results were analyzed by PP with the help JS. Manuscript was written by PP with the help from JS and manuscript revision was done by PP.

## Conflict of Interest Statement

The authors declare that the research was conducted in the absence of any commercial or financial relationships that could be construed as a potential conflict of interest. The reviewer, YB, and handling editor declared their shared affiliation, and the handling editor states that the process nevertheless met the standards of a fair and objective review.
